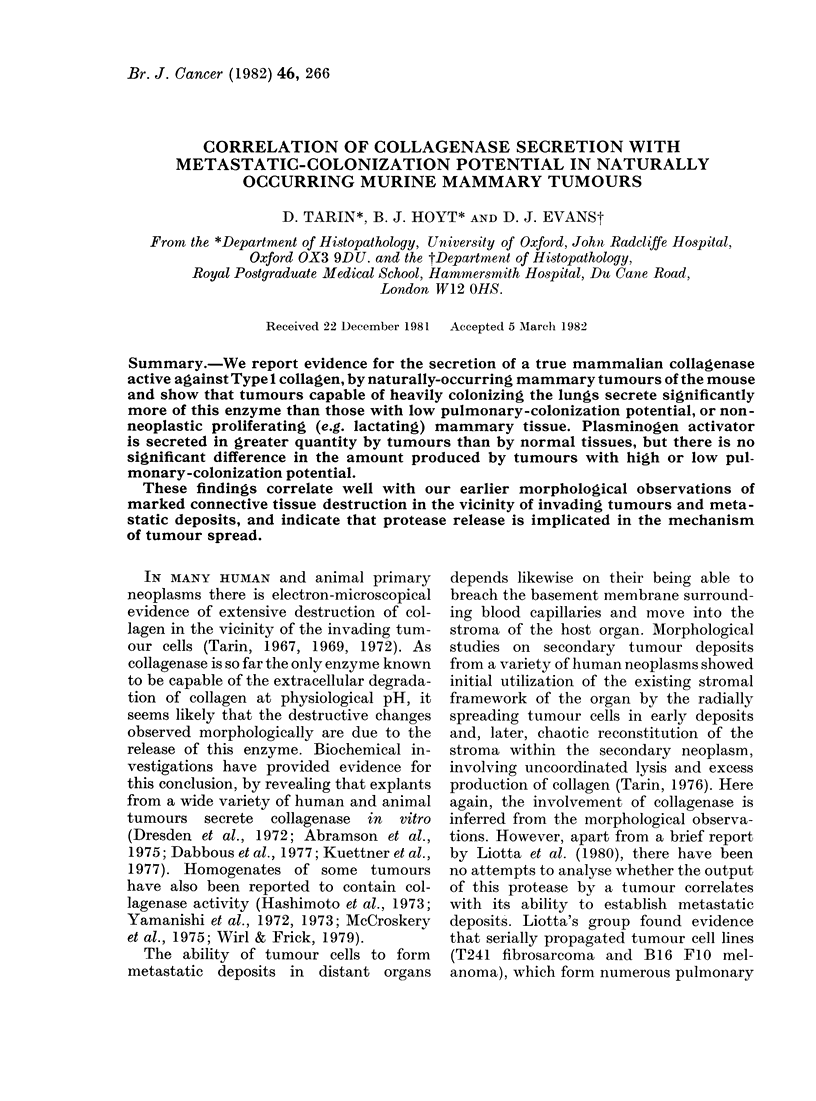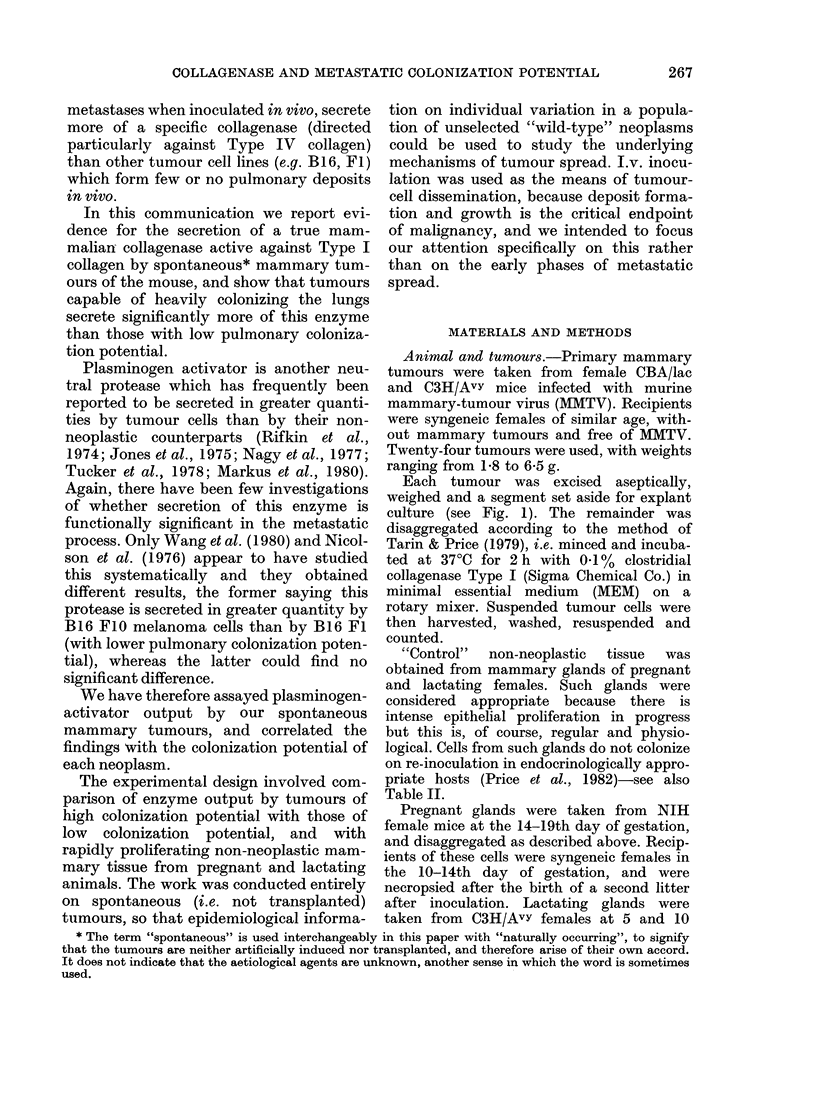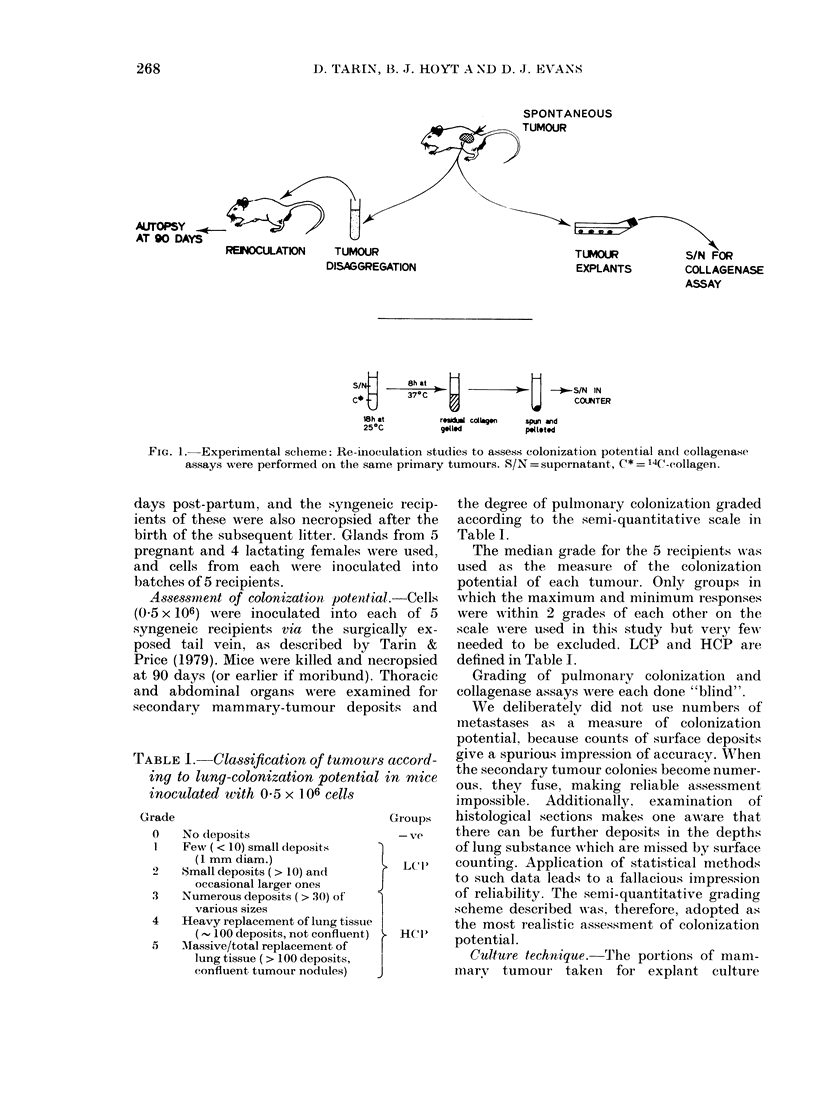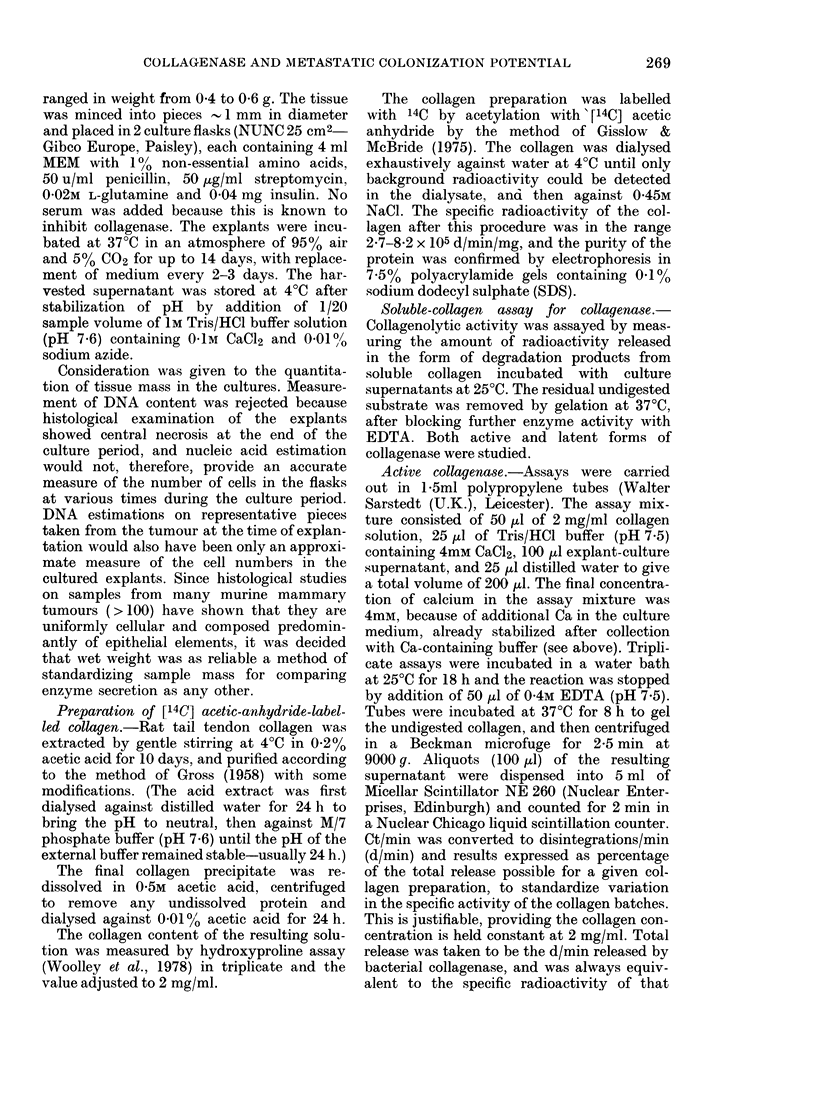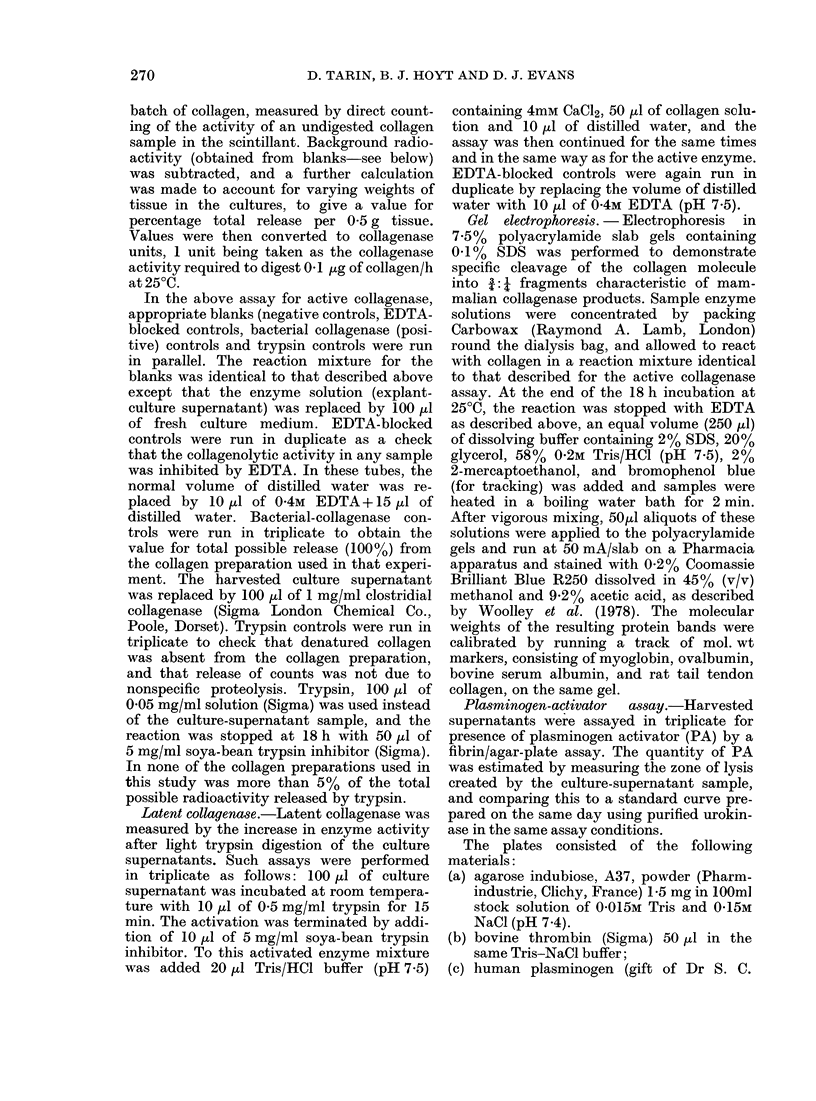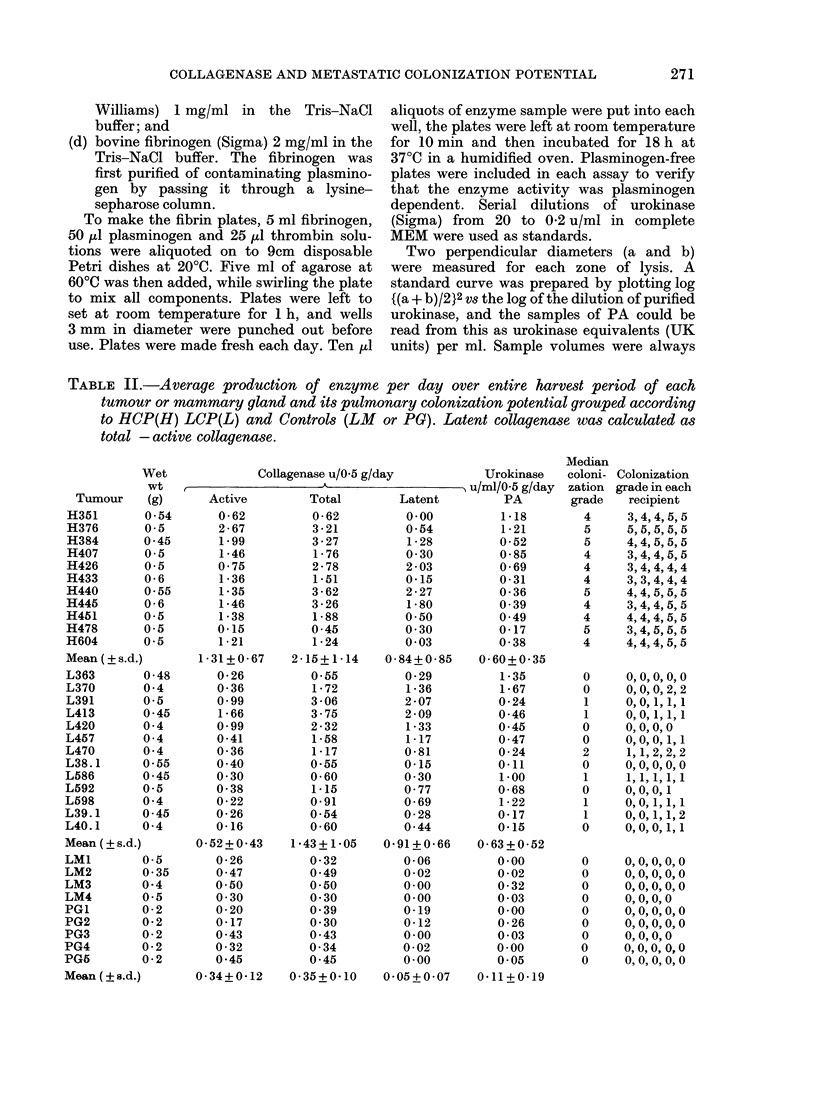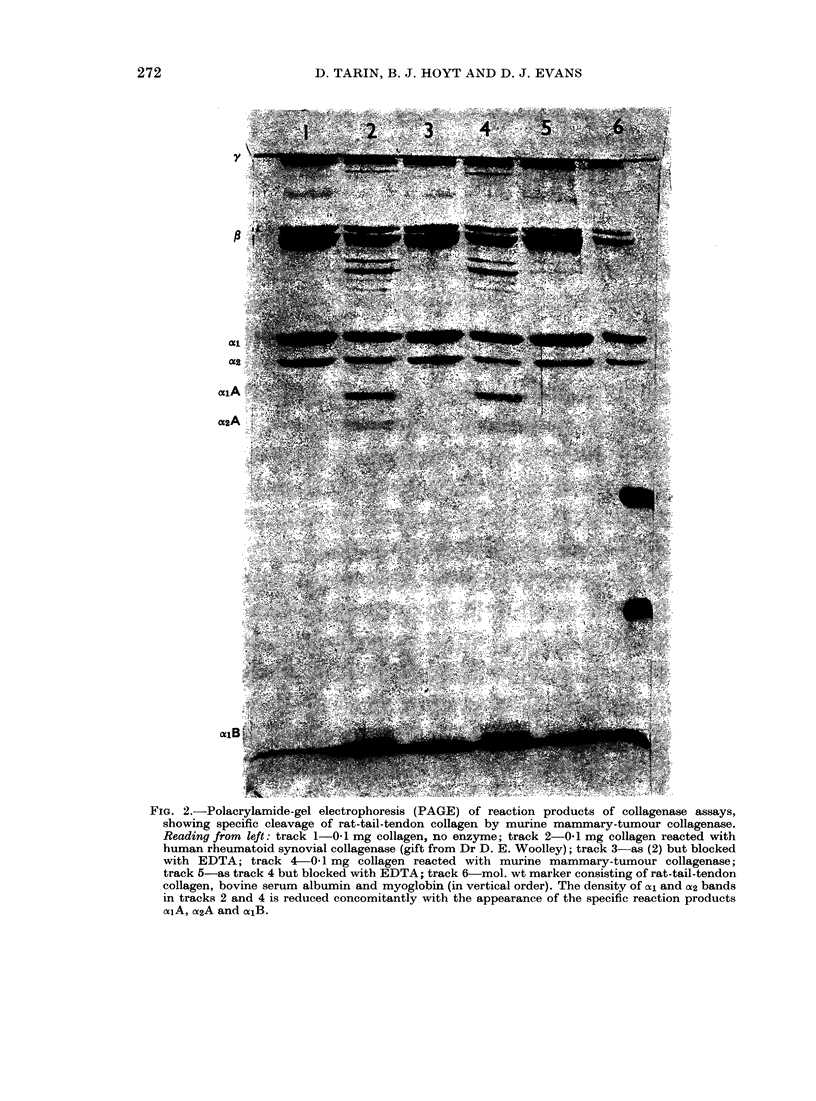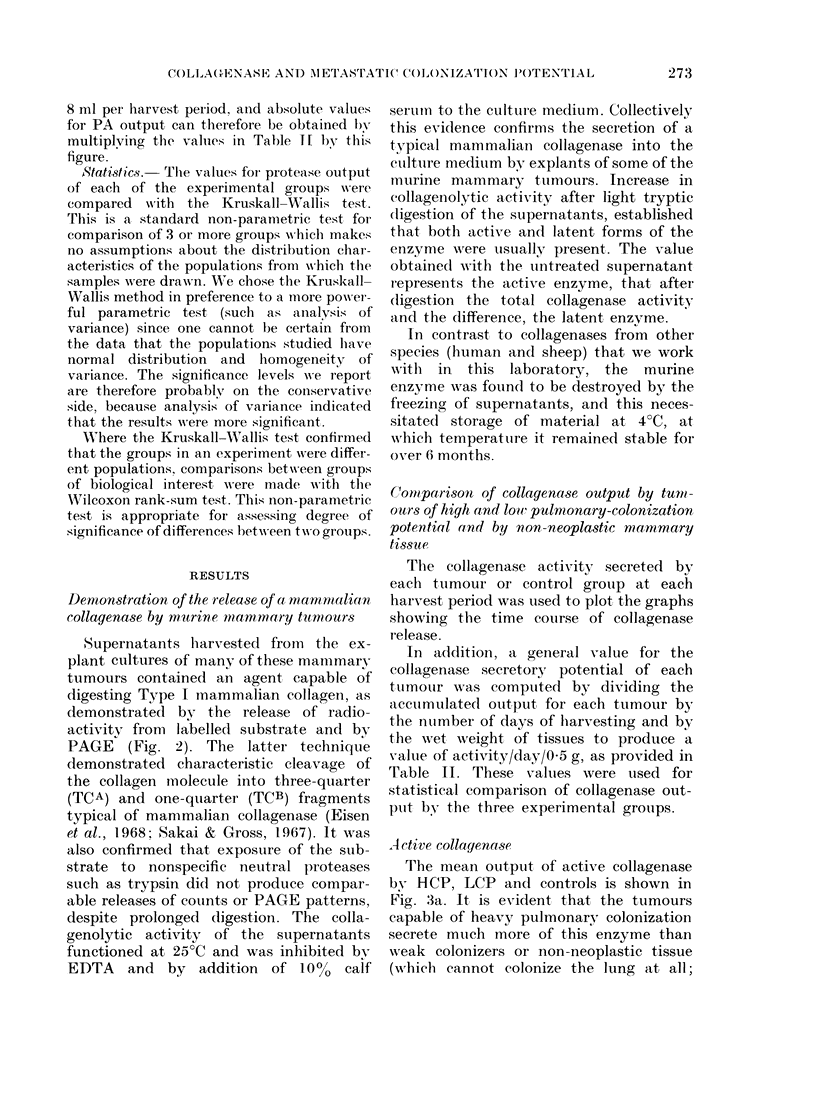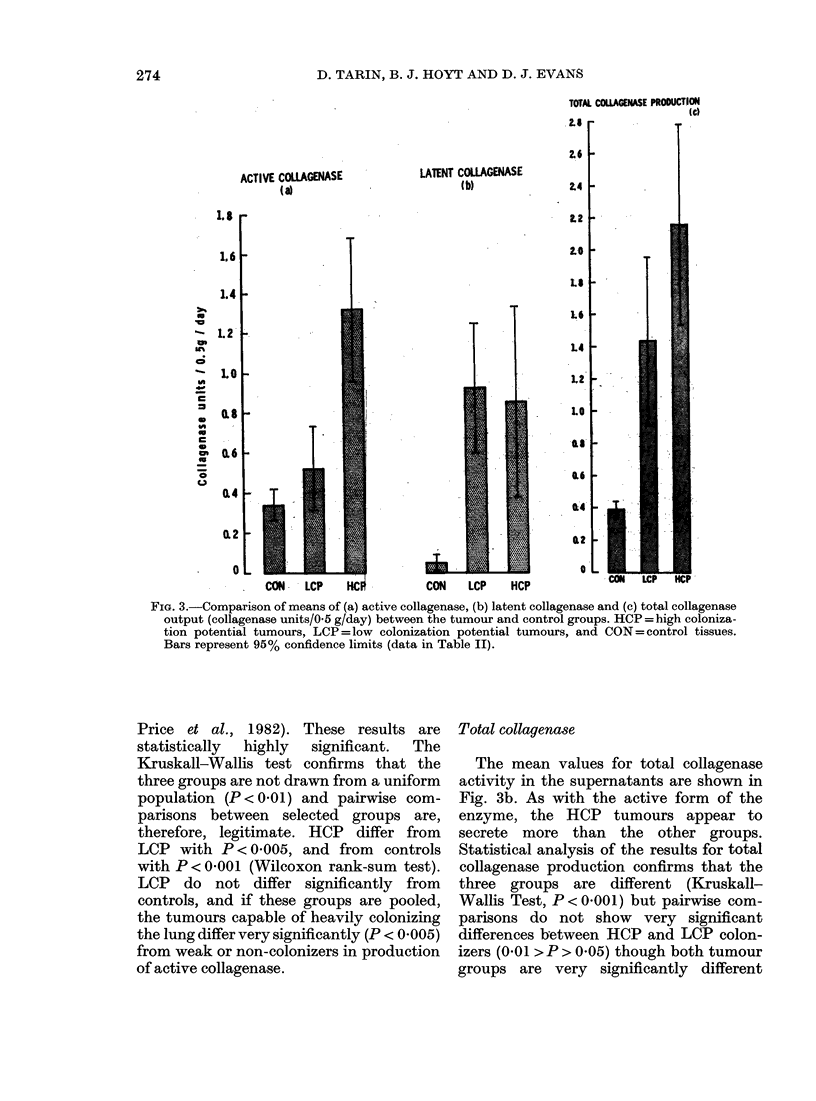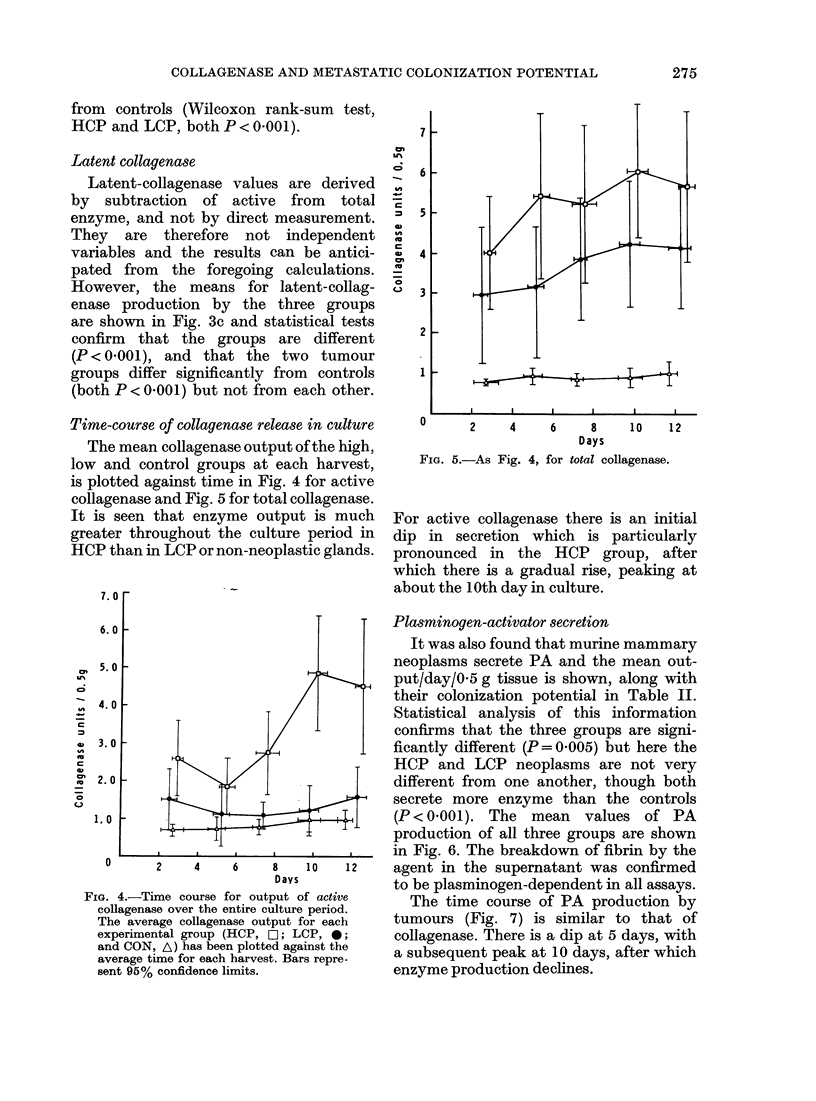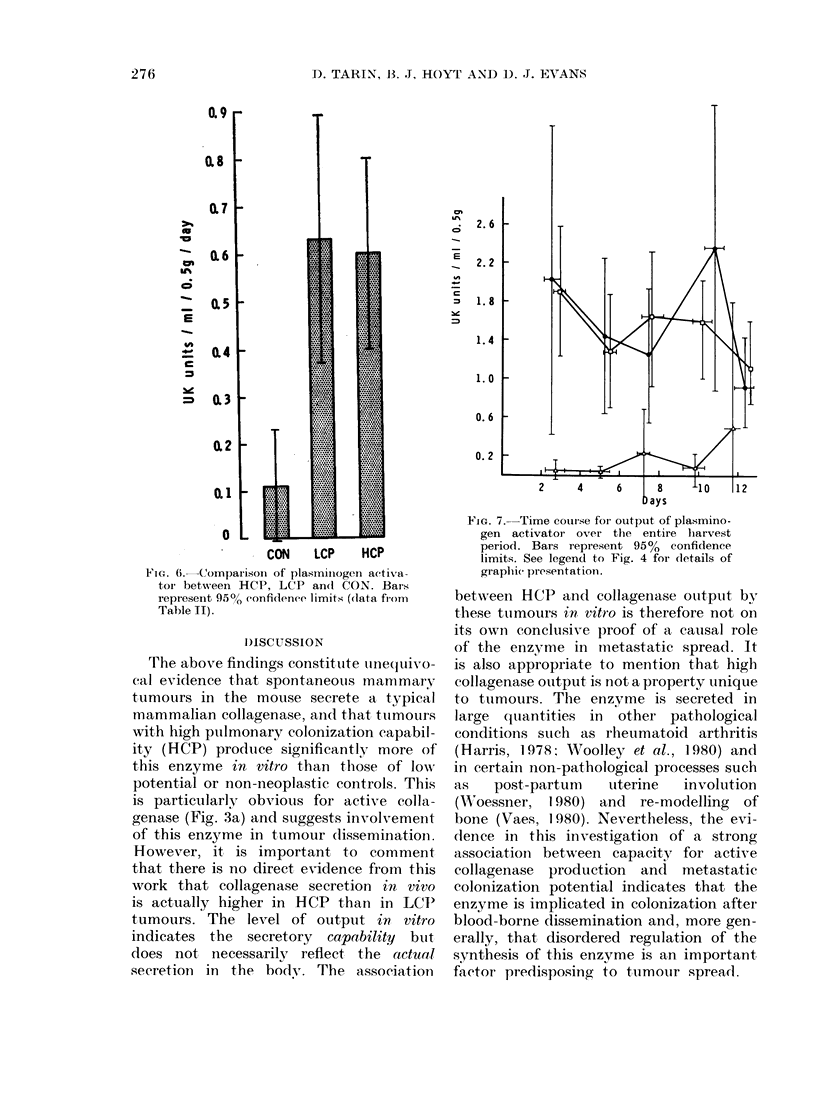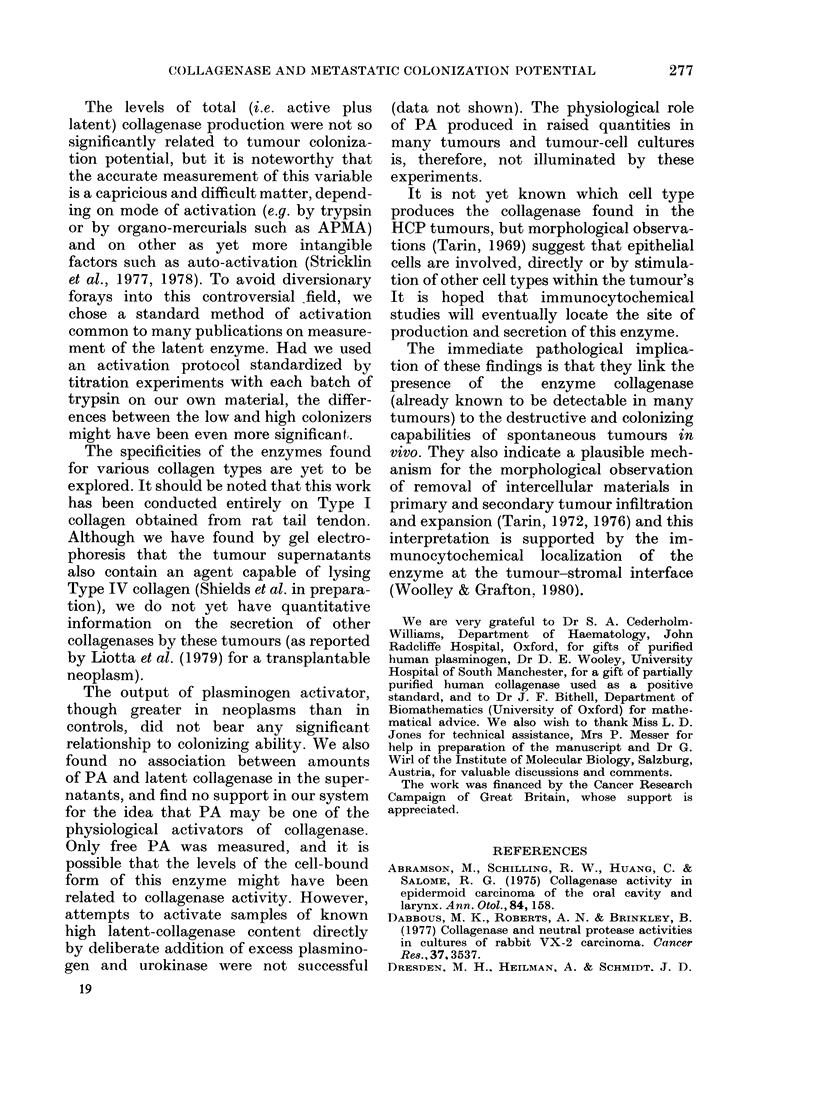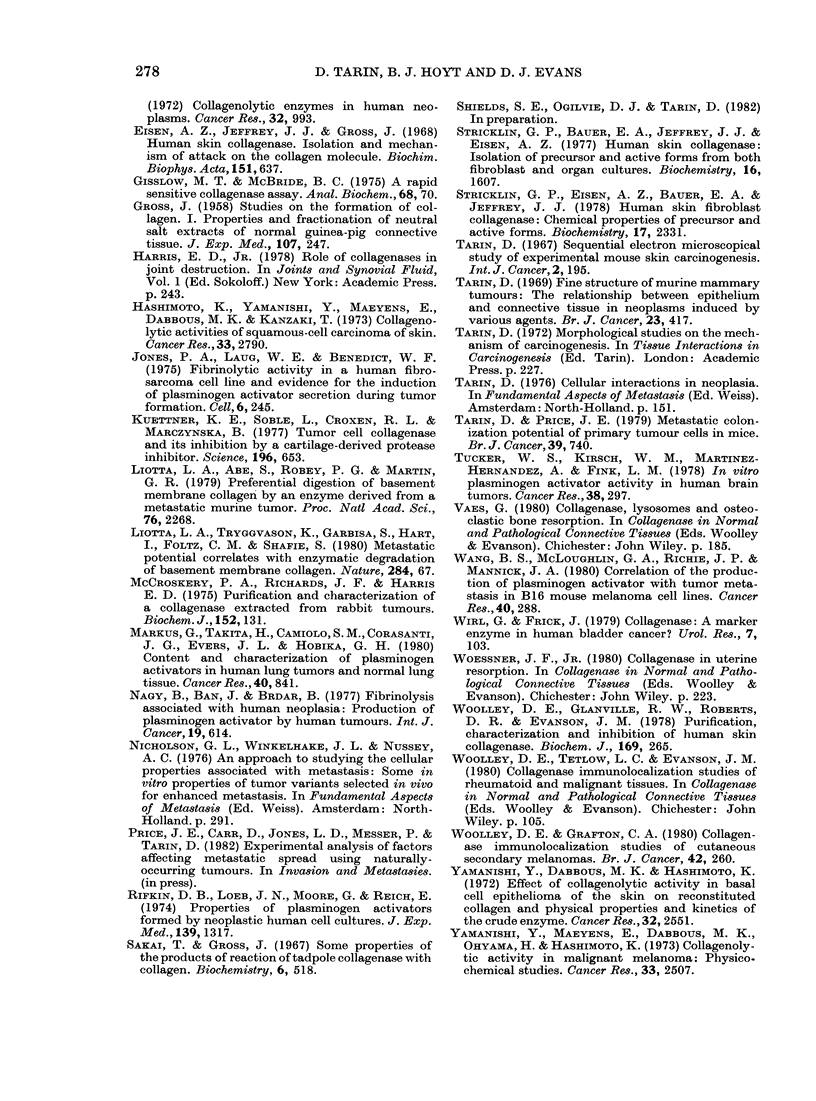# Correlation of collagenase secretion with metastatic-colonization potential in naturally occurring murine mammary tumours.

**DOI:** 10.1038/bjc.1982.192

**Published:** 1982-08

**Authors:** D. Tarin, B. J. Hoyt, D. J. Evans

## Abstract

**Images:**


*Br. J. Cancer* (1982) **46**, 266

### CORRELATION OF COLLAGENASE SECRETION WITH METASTATIC-COLONIZATION POTENTIAL IN NATURALLY OCCURRING MURINE MAMMARY TUMOURS

D. TARIN*, B. J. HOYT* AND D. J. EVANS†

*From the *Department of Histopathology, University of Oxford, John Radcliffe Hospital, Oxford OX3 9DU. and the †Department of Histopathology, Royal Postgraduate Medical School, Hammersmith Hospital, Du Cane Road, London W12 0HS.*

Received 22 December 1981 Accepted 5 March 1982

**Summary.—We report evidence for the secretion of a true mammalian collagenase active against Type 1 collagen, by naturally-occurring mammary tumours of the mouse and show that tumours capable of heavily colonizing the lungs secrete significantly more of this enzyme than those with low pulmonary-colonization potential, or non-neoplastic proliferating (*e.g.* lactating) mammary tissue. Plasminogen activator is secreted in greater quantity by tumours than by normal tissues, but there is no significant difference in the amount produced by tumours with high or low pulmonary-colonization potential.**

**These findings correlate well with our earlier morphological observations of marked connective tissue destruction in the vicinity of invading tumours and metastatic deposits, and indicate that protease release is implicated in the mechanism of tumour spread.**

IN MANY HUMAN and animal primary neoplasms there is electron-microscopical evidence of extensive destruction of collagen in the vicinity of the invading tumour cells (Tarin, 1967, 1969, 1972). As collagenase is so far the only enzyme known to be capable of the extracellular degradation of collagen at physiological pH, it seems likely that the destructive changes observed morphologically are due to the release of this enzyme. Biochemical investigations have provided evidence for this conclusion, by revealing that explants from a wide variety of human and animal tumours secrete collagenase *in vitro* (Dresden *et al.*, 1972; Abramson *et al.*, 1975; Dabbous *et al.*, 1977; Kuettner *et al.*, 1977). Homogenates of some tumours have also been reported to contain collagenase activity (Hashimoto *et al.*, 1973; Yamanishi *et al.*, 1972, 1973; McCroskery *et al.*, 1975; Wirl & Frick, 1979).

The ability of tumour cells to form metastatic deposits in distant organs depends likewise on their being able to breach the basement membrane surrounding blood capillaries and move into the stroma of the host organ. Morphological studies on secondary tumour deposits from a variety of human neoplasms showed initial utilization of the existing stromal framework of the organ by the radially spreading tumour cells in early deposits and, later, chaotic reconstitution of the stroma within the secondary neoplasm, involving uncoordinated lysis and excess production of collagen (Tarin, 1976). Here again, the involvement of collagenase is inferred from the morphological observations. However, apart from a brief report by Liotta *et al.* (1980), there have been no attempts to analyse whether the output of this protease by a tumour correlates with its ability to establish metastatic deposits. Liotta's group found evidence that serially propagated tumour cell lines (T241 fibrosarcoma and B16 F10 melanoma), which form numerous pulmonary metastases when inoculated *in vivo*, secrete more of a specific collagenase (directed particularly against Type IV collagen) than other tumour cell lines (*e.g.* B16, F1) which form few or no pulmonary deposits *in vivo*.

In this communication we report evidence for the secretion of a true mammalian collagenase active against Type I collagen by spontaneous* mammary tumours of the mouse, and show that tumours capable of heavily colonizing the lungs secrete significantly more of this enzyme than those with low pulmonary colonization potential.

Plasminogen activator is another neutral protease which has frequently been reported to be secreted in greater quantities by tumour cells than by their non-neoplastic counterparts (Rifkin *et al.*, 1974; Jones *et al.*, 1975; Nagy *et al.*, 1977; Tucker *et al.*, 1978; Markus *et al.*, 1980). Again, there have been few investigations of whether secretion of this enzyme is functionally significant in the metastatic process. Only Wang *et al.* (1980) and Nicolson *et al.* (1976) appear to have studied this systematically and they obtained different results, the former saying this protease is secreted in greater quantity by B16 F10 melanoma cells than by B16 F1 (with lower pulmonary colonization potential), whereas the latter could find no significant difference.

We have therefore assayed plasminogen-activator output by our spontaneous mammary tumours, and correlated the findings with the colonization potential of each neoplasm.

The experimental design involved comparison of enzyme output by tumours of high colonization potential with those of low colonization potential, and with rapidly proliferating non-neoplastic mammary tissue from pregnant and lactating animals. The work was conducted entirely on spontaneous (*i.e.* not transplanted) tumours, so that epidemiological information on individual variation in a population of unselected "wild-type" neoplasms could be used to study the underlying mechanisms of tumour spread. I.v. inoculation was used as the means of tumour-cell dissemination, because deposit formation and growth is the critical endpoint of malignancy, and we intended to focus our attention specifically on this rather than on the early phases of metastatic spread.

### MATERIALS AND METHODS

*Animal and tumours.*—Primary mammary tumours were taken from female CBA/lac and C3H/$A^{vy}$ mice infected with murine mammary-tumour virus (MMTV). Recipients were syngeneic females of similar age, without mammary tumours and free of MMTV. Twenty-four tumours were used, with weights ranging from 1·8 to 6·5 g.

Each tumour was excised aseptically, weighed and a segment set aside for explant culture (see Fig. 1). The remainder was disaggregated according to the method of Tarin & Price (1979), *i.e.* minced and incubated at 37°C for 2 h with 0·1% clostridial collagenase Type I (Sigma Chemical Co.) in minimal essential medium (MEM) on a rotary mixer. Suspended tumour cells were then harvested, washed, resuspended and counted.

"Control" non-neoplastic tissue was obtained from mammary glands of pregnant and lactating females. Such glands were considered appropriate because there is intense epithelial proliferation in progress but this is, of course, regular and physiological. Cells from such glands do not colonize on re-inoculation in endocrinologically appropriate hosts (Price *et al.*, 1982)—see also Table II.

Pregnant glands were taken from NIH female mice at the 14–19th day of gestation, and disaggregated as described above. Recipients of these cells were syngeneic females in the 10–14th day of gestation, and were necropsied after the birth of a second litter after inoculation. Lactating glands were taken from C3H/$A^{vy}$ females at 5 and 10

* The term "spontaneous" is used interchangeably in this paper with "naturally occurring", to signify that the tumours are neither artificially induced nor transplanted, and therefore arise of their own accord. It does not indicate that the aetiological agents are unknown, another sense in which the word is sometimes used.

FIG. 1.—Experimental scheme: Re-inoculation studies to assess colonization potential and collagenase assays were performed on the same primary tumours. S/N = supernatant, C* = $^{14}$C-collagen.

days post-partum, and the syngeneic recipients of these were also necropsied after the birth of the subsequent litter. Glands from 5 pregnant and 4 lactating females were used, and cells from each were inoculated into batches of 5 recipients.

*Assessment of colonization potential.*—Cells ($0{\cdot}5 \times 10^6$) were inoculated into each of 5 syngeneic recipients *via* the surgically exposed tail vein, as described by Tarin & Price (1979). Mice were killed and necropsied at 90 days (or earlier if moribund). Thoracic and abdominal organs were examined for secondary mammary-tumour deposits and

TABLE I.—*Classification of tumours according to lung-colonization potential in mice inoculated with* $0{\cdot}5 \times 10^6$ *cells*

| Grade | | Groups |
|---|---|---|
| 0 | No deposits | −ve |
| 1 | Few ( < 10) small deposits (1 mm diam.) | LCP |
| 2 | Small deposits ( > 10) and occasional larger ones | LCP |
| 3 | Numerous deposits ( > 30) of various sizes | HCP |
| 4 | Heavy replacement of lung tissue ( ~ 100 deposits, not confluent) | HCP |
| 5 | Massive/total replacement of lung tissue ( > 100 deposits, confluent tumour nodules) | HCP |

the degree of pulmonary colonization graded according to the semi-quantitative scale in Table I.

The median grade for the 5 recipients was used as the measure of the colonization potential of each tumour. Only groups in which the maximum and minimum responses were within 2 grades of each other on the scale were used in this study but very few needed to be excluded. LCP and HCP are defined in Table I.

Grading of pulmonary colonization and collagenase assays were each done "blind".

We deliberately did not use numbers of metastases as a measure of colonization potential, because counts of surface deposits give a spurious impression of accuracy. When the secondary tumour colonies become numerous, they fuse, making reliable assessment impossible. Additionally, examination of histological sections makes one aware that there can be further deposits in the depths of lung substance which are missed by surface counting. Application of statistical methods to such data leads to a fallacious impression of reliability. The semi-quantitative grading scheme described was, therefore, adopted as the most realistic assessment of colonization potential.

*Culture technique.*—The portions of mammary tumour taken for explant culture ranged in weight from 0·4 to 0·6 g. The tissue was minced into pieces ~1 mm in diameter and placed in 2 culture flasks (NUNC 25 $cm^2$—Gibco Europe, Paisley), each containing 4 ml MEM with 1% non-essential amino acids, 50 u/ml penicillin, 50 μg/ml streptomycin, 0·02M L-glutamine and 0·04 mg insulin. No serum was added because this is known to inhibit collagenase. The explants were incubated at 37°C in an atmosphere of 95% air and 5% $CO_2$ for up to 14 days, with replacement of medium every 2–3 days. The harvested supernatant was stored at 4°C after stabilization of pH by addition of 1/20 sample volume of 1M Tris/HCl buffer solution (pH 7·6) containing 0·1M $CaCl_2$ and 0·01% sodium azide.

Consideration was given to the quantitation of tissue mass in the cultures. Measurement of DNA content was rejected because histological examination of the explants showed central necrosis at the end of the culture period, and nucleic acid estimation would not, therefore, provide an accurate measure of the number of cells in the flasks at various times during the culture period. DNA estimations on representative pieces taken from the tumour at the time of explantation would also have been only an approximate measure of the cell numbers in the cultured explants. Since histological studies on samples from many murine mammary tumours (>100) have shown that they are uniformly cellular and composed predominantly of epithelial elements, it was decided that wet weight was as reliable a method of standardizing sample mass for comparing enzyme secretion as any other.

*Preparation of [$^{14}C$] acetic-anhydride-labelled collagen.*—Rat tail tendon collagen was extracted by gentle stirring at 4°C in 0·2% acetic acid for 10 days, and purified according to the method of Gross (1958) with some modifications. (The acid extract was first dialysed against distilled water for 24 h to bring the pH to neutral, then against M/7 phosphate buffer (pH 7·6) until the pH of the external buffer remained stable—usually 24 h.)

The final collagen precipitate was redissolved in 0·5M acetic acid, centrifuged to remove any undissolved protein and dialysed against 0·01% acetic acid for 24 h.

The collagen content of the resulting solution was measured by hydroxyproline assay (Woolley *et al.*, 1978) in triplicate and the value adjusted to 2 mg/ml.

The collagen preparation was labelled with $^{14}C$ by acetylation with [$^{14}C$] acetic anhydride by the method of Gisslow & McBride (1975). The collagen was dialysed exhaustively against water at 4°C until only background radioactivity could be detected in the dialysate, and then against 0·45M NaCl. The specific radioactivity of the collagen after this procedure was in the range $2{\cdot}7$–$8{\cdot}2 \times 10^5$ d/min/mg, and the purity of the protein was confirmed by electrophoresis in 7·5% polyacrylamide gels containing 0·1% sodium dodecyl sulphate (SDS).

*Soluble-collagen assay for collagenase.*—Collagenolytic activity was assayed by measuring the amount of radioactivity released in the form of degradation products from soluble collagen incubated with culture supernatants at 25°C. The residual undigested substrate was removed by gelation at 37°C, after blocking further enzyme activity with EDTA. Both active and latent forms of collagenase were studied.

*Active collagenase.*—Assays were carried out in 1·5ml polypropylene tubes (Walter Sarstedt (U.K.), Leicester). The assay mixture consisted of 50 μl of 2 mg/ml collagen solution, 25 μl of Tris/HCl buffer (pH 7·5) containing 4mM $CaCl_2$, 100 μl explant-culture supernatant, and 25 μl distilled water to give a total volume of 200 μl. The final concentration of calcium in the assay mixture was 4mM, because of additional Ca in the culture medium, already stabilized after collection with Ca-containing buffer (see above). Triplicate assays were incubated in a water bath at 25°C for 18 h and the reaction was stopped by addition of 50 μl of 0·4M EDTA (pH 7·5). Tubes were incubated at 37°C for 8 h to gel the undigested collagen, and then centrifuged in a Beckman microfuge for 2·5 min at 9000 *g*. Aliquots (100 μl) of the resulting supernatant were dispensed into 5 ml of Micellar Scintillator NE 260 (Nuclear Enterprises, Edinburgh) and counted for 2 min in a Nuclear Chicago liquid scintillation counter. Ct/min was converted to disintegrations/min (d/min) and results expressed as percentage of the total release possible for a given collagen preparation, to standardize variation in the specific activity of the collagen batches. This is justifiable, providing the collagen concentration is held constant at 2 mg/ml. Total release was taken to be the d/min released by bacterial collagenase, and was always equivalent to the specific radioactivity of that batch of collagen, measured by direct counting of the activity of an undigested collagen sample in the scintillant. Background radioactivity (obtained from blanks—see below) was subtracted, and a further calculation was made to account for varying weights of tissue in the cultures, to give a value for percentage total release per 0·5 g tissue. Values were then converted to collagenase units, 1 unit being taken as the collagenase activity required to digest 0·1 $\mu$g of collagen/h at 25°C.

In the above assay for active collagenase, appropriate blanks (negative controls, EDTA-blocked controls, bacterial collagenase (positive) controls and trypsin controls were run in parallel. The reaction mixture for the blanks was identical to that described above except that the enzyme solution (explant-culture supernatant) was replaced by 100 $\mu$l of fresh culture medium. EDTA-blocked controls were run in duplicate as a check that the collagenolytic activity in any sample was inhibited by EDTA. In these tubes, the normal volume of distilled water was replaced by 10 $\mu$l of 0·4M EDTA + 15 $\mu$l of distilled water. Bacterial-collagenase controls were run in triplicate to obtain the value for total possible release (100%) from the collagen preparation used in that experiment. The harvested culture supernatant was replaced by 100 $\mu$l of 1 mg/ml clostridial collagenase (Sigma London Chemical Co., Poole, Dorset). Trypsin controls were run in triplicate to check that denatured collagen was absent from the collagen preparation, and that release of counts was not due to nonspecific proteolysis. Trypsin, 100 $\mu$l of 0·05 mg/ml solution (Sigma) was used instead of the culture-supernatant sample, and the reaction was stopped at 18 h with 50 $\mu$l of 5 mg/ml soya-bean trypsin inhibitor (Sigma). In none of the collagen preparations used in this study was more than 5% of the total possible radioactivity released by trypsin.

*Latent collagenase.*—Latent collagenase was measured by the increase in enzyme activity after light trypsin digestion of the culture supernatants. Such assays were performed in triplicate as follows: 100 $\mu$l of culture supernatant was incubated at room temperature with 10 $\mu$l of 0·5 mg/ml trypsin for 15 min. The activation was terminated by addition of 10 $\mu$l of 5 mg/ml soya-bean trypsin inhibitor. To this activated enzyme mixture was added 20 $\mu$l Tris/HCl buffer (pH 7·5) containing 4mM $CaCl_2$, 50 $\mu$l of collagen solution and 10 $\mu$l of distilled water, and the assay was then continued for the same times and in the same way as for the active enzyme. EDTA-blocked controls were again run in duplicate by replacing the volume of distilled water with 10 $\mu$l of 0·4M EDTA (pH 7·5).

*Gel electrophoresis.* — Electrophoresis in 7·5% polyacrylamide slab gels containing 0·1% SDS was performed to demonstrate specific cleavage of the collagen molecule into $\frac{3}{4}$:$\frac{1}{4}$ fragments characteristic of mammalian collagenase products. Sample enzyme solutions were concentrated by packing Carbowax (Raymond A. Lamb, London) round the dialysis bag, and allowed to react with collagen in a reaction mixture identical to that described for the active collagenase assay. At the end of the 18 h incubation at 25°C, the reaction was stopped with EDTA as described above, an equal volume (250 $\mu$l) of dissolving buffer containing 2% SDS, 20% glycerol, 58% 0·2M Tris/HCl (pH 7·5), 2% 2-mercaptoethanol, and bromophenol blue (for tracking) was added and samples were heated in a boiling water bath for 2 min. After vigorous mixing, 50$\mu$l aliquots of these solutions were applied to the polyacrylamide gels and run at 50 mA/slab on a Pharmacia apparatus and stained with 0·2% Coomassie Brilliant Blue R250 dissolved in 45% (v/v) methanol and 9·2% acetic acid, as described by Woolley *et al.* (1978). The molecular weights of the resulting protein bands were calibrated by running a track of mol. wt markers, consisting of myoglobin, ovalbumin, bovine serum albumin, and rat tail tendon collagen, on the same gel.

*Plasminogen-activator assay.*—Harvested supernatants were assayed in triplicate for presence of plasminogen activator (PA) by a fibrin/agar-plate assay. The quantity of PA was estimated by measuring the zone of lysis created by the culture-supernatant sample, and comparing this to a standard curve prepared on the same day using purified urokinase in the same assay conditions.

The plates consisted of the following materials:

(a) agarose indubiose, A37, powder (Pharmindustrie, Clichy, France) 1·5 mg in 100ml stock solution of 0·015M Tris and 0·15M NaCl (pH 7·4).
(b) bovine thrombin (Sigma) 50 $\mu$l in the same Tris–NaCl buffer;
(c) human plasminogen (gift of Dr S. C.

Williams) 1 mg/ml in the Tris–NaCl buffer; and

(d) bovine fibrinogen (Sigma) 2 mg/ml in the Tris–NaCl buffer. The fibrinogen was first purified of contaminating plasminogen by passing it through a lysine–sepharose column.

To make the fibrin plates, 5 ml fibrinogen, 50 μl plasminogen and 25 μl thrombin solutions were aliquoted on to 9cm disposable Petri dishes at 20°C. Five ml of agarose at 60°C was then added, while swirling the plate to mix all components. Plates were left to set at room temperature for 1 h, and wells 3 mm in diameter were punched out before use. Plates were made fresh each day. Ten μl aliquots of enzyme sample were put into each well, the plates were left at room temperature for 10 min and then incubated for 18 h at 37°C in a humidified oven. Plasminogen-free plates were included in each assay to verify that the enzyme activity was plasminogen dependent. Serial dilutions of urokinase (Sigma) from 20 to 0·2 u/ml in complete MEM were used as standards.

Two perpendicular diameters (a and b) were measured for each zone of lysis. A standard curve was prepared by plotting log $\{(a+b)/2\}^2$ *vs* the log of the dilution of purified urokinase, and the samples of PA could be read from this as urokinase equivalents (UK units) per ml. Sample volumes were always

TABLE II.—*Average production of enzyme per day over entire harvest period of each tumour or mammary gland and its pulmonary colonization potential grouped according to HCP(H) LCP(L) and Controls (LM or PG). Latent collagenase was calculated as total − active collagenase.*

| Tumour | Wet wt (g) | Collagenase u/0·5 g/day Active | Total | Latent | Urokinase u/ml/0·5 g/day PA | Median colonization grade | Colonization grade in each recipient |
|---|---|---|---|---|---|---|---|
| H351 | 0·54 | 0·62 | 0·62 | 0·00 | 1·18 | 4 | 3, 4, 4, 5, 5 |
| H376 | 0·5 | 2·67 | 3·21 | 0·54 | 1·21 | 5 | 5, 5, 5, 5, 5 |
| H384 | 0·45 | 1·99 | 3·27 | 1·28 | 0·52 | 5 | 4, 4, 5, 5, 5 |
| H407 | 0·5 | 1·46 | 1·76 | 0·30 | 0·85 | 4 | 3, 4, 4, 5, 5 |
| H426 | 0·5 | 0·75 | 2·78 | 2·03 | 0·69 | 4 | 3, 4, 4, 4, 4 |
| H433 | 0·6 | 1·36 | 1·51 | 0·15 | 0·31 | 4 | 3, 3, 4, 4, 4 |
| H440 | 0·55 | 1·35 | 3·62 | 2·27 | 0·36 | 5 | 4, 4, 5, 5, 5 |
| H445 | 0·6 | 1·46 | 3·26 | 1·80 | 0·39 | 4 | 3, 4, 4, 5, 5 |
| H451 | 0·5 | 1·38 | 1·88 | 0·50 | 0·49 | 4 | 4, 4, 4, 5, 5 |
| H478 | 0·5 | 0·15 | 0·45 | 0·30 | 0·17 | 5 | 3, 4, 5, 5, 5 |
| H604 | 0·5 | 1·21 | 1·24 | 0·03 | 0·38 | 4 | 4, 4, 4, 5, 5 |
| Mean (±s.d.) | | 1·31±0·67 | 2·15±1·14 | 0·84±0·85 | 0·60±0·35 | | |
| L363 | 0·48 | 0·26 | 0·55 | 0·29 | 1·35 | 0 | 0, 0, 0, 0, 0 |
| L370 | 0·4 | 0·36 | 1·72 | 1·36 | 1·67 | 0 | 0, 0, 0, 2, 2 |
| L391 | 0·5 | 0·99 | 3·06 | 2·07 | 0·24 | 1 | 0, 0, 1, 1, 1 |
| L413 | 0·45 | 1·66 | 3·75 | 2·09 | 0·46 | 1 | 0, 0, 1, 1, 1 |
| L420 | 0·4 | 0·99 | 2·32 | 1·33 | 0·45 | 0 | 0, 0, 0, 0 |
| L457 | 0·4 | 0·41 | 1·58 | 1·17 | 0·47 | 0 | 0, 0, 0, 1, 1 |
| L470 | 0·4 | 0·36 | 1·17 | 0·81 | 0·24 | 2 | 1, 1, 2, 2, 2 |
| L38.1 | 0·55 | 0·40 | 0·55 | 0·15 | 0·11 | 0 | 0, 0, 0, 0, 0 |
| L586 | 0·45 | 0·30 | 0·60 | 0·30 | 1·00 | 1 | 1, 1, 1, 1, 1 |
| L592 | 0·5 | 0·38 | 1·15 | 0·77 | 0·68 | 0 | 0, 0, 0, 1 |
| L598 | 0·4 | 0·22 | 0·91 | 0·69 | 1·22 | 1 | 0, 0, 1, 1, 1 |
| L39.1 | 0·45 | 0·26 | 0·54 | 0·28 | 0·17 | 1 | 0, 0, 1, 1, 2 |
| L40.1 | 0·4 | 0·16 | 0·60 | 0·44 | 0·15 | 0 | 0, 0, 0, 1, 1 |
| Mean (±s.d.) | | 0·52±0·43 | 1·43±1·05 | 0·91±0·66 | 0·63±0·52 | | |
| LM1 | 0·5 | 0·26 | 0·32 | 0·06 | 0·00 | 0 | 0, 0, 0, 0, 0 |
| LM2 | 0·35 | 0·47 | 0·49 | 0·02 | 0·02 | 0 | 0, 0, 0, 0, 0 |
| LM3 | 0·4 | 0·50 | 0·50 | 0·00 | 0·32 | 0 | 0, 0, 0, 0, 0 |
| LM4 | 0·5 | 0·30 | 0·30 | 0·00 | 0·03 | 0 | 0, 0, 0, 0 |
| PG1 | 0·2 | 0·20 | 0·39 | 0·19 | 0·00 | 0 | 0, 0, 0, 0, 0 |
| PG2 | 0·2 | 0·17 | 0·30 | 0·12 | 0·26 | 0 | 0, 0, 0, 0, 0 |
| PG3 | 0·2 | 0·43 | 0·43 | 0·00 | 0·03 | 0 | 0, 0, 0, 0 |
| PG4 | 0·2 | 0·32 | 0·34 | 0·02 | 0·00 | 0 | 0, 0, 0, 0, 0 |
| PG5 | 0·2 | 0·45 | 0·45 | 0·00 | 0·05 | 0 | 0, 0, 0, 0, 0 |
| Mean (±s.d.) | | 0·34±0·12 | 0·35±0·10 | 0·05±0·07 | 0·11±0·19 | | |

FIG. 2.—Polacrylamide-gel electrophoresis (PAGE) of reaction products of collagenase assays, showing specific cleavage of rat-tail-tendon collagen by murine mammary-tumour collagenase. *Reading from left:* track 1—0·1 mg collagen, no enzyme; track 2—0·1 mg collagen reacted with human rheumatoid synovial collagenase (gift from Dr D. E. Woolley); track 3—as (2) but blocked with EDTA; track 4—0·1 mg collagen reacted with murine mammary-tumour collagenase; track 5—as track 4 but blocked with EDTA; track 6—mol. wt marker consisting of rat-tail-tendon collagen, bovine serum albumin and myoglobin (in vertical order). The density of $\alpha_1$ and $\alpha_2$ bands in tracks 2 and 4 is reduced concomitantly with the appearance of the specific reaction products $\alpha_1$A, $\alpha_2$A and $\alpha_1$B.

8 ml per harvest period, and absolute values for PA output can therefore be obtained by multiplying the values in Table II by this figure.

*Statistics.*— The values for protease output of each of the experimental groups were compared with the Kruskall–Wallis test. This is a standard non-parametric test for comparison of 3 or more groups which makes no assumptions about the distribution characteristics of the populations from which the samples were drawn. We chose the Kruskall–Wallis method in preference to a more powerful parametric test (such as analysis of variance) since one cannot be certain from the data that the populations studied have normal distribution and homogeneity of variance. The significance levels we report are therefore probably on the conservative side, because analysis of variance indicated that the results were more significant.

Where the Kruskall–Wallis test confirmed that the groups in an experiment were different populations, comparisons between groups of biological interest were made with the Wilcoxon rank-sum test. This non-parametric test is appropriate for assessing degree of significance of differences between two groups.

### RESULTS

### *Demonstration of the release of a mammalian collagenase by murine mammary tumours*

Supernatants harvested from the explant cultures of many of these mammary tumours contained an agent capable of digesting Type I mammalian collagen, as demonstrated by the release of radioactivity from labelled substrate and by PAGE (Fig. 2). The latter technique demonstrated characteristic cleavage of the collagen molecule into three-quarter ($TC^A$) and one-quarter ($TC^B$) fragments typical of mammalian collagenase (Eisen *et al.*, 1968; Sakai & Gross, 1967). It was also confirmed that exposure of the substrate to nonspecific neutral proteases such as trypsin did not produce comparable releases of counts or PAGE patterns, despite prolonged digestion. The collagenolytic activity of the supernatants functioned at 25°C and was inhibited by EDTA and by addition of 10% calf serum to the culture medium. Collectively this evidence confirms the secretion of a typical mammalian collagenase into the culture medium by explants of some of the murine mammary tumours. Increase in collagenolytic activity after light tryptic digestion of the supernatants, established that both active and latent forms of the enzyme were usually present. The value obtained with the untreated supernatant represents the active enzyme, that after digestion the total collagenase activity and the difference, the latent enzyme.

In contrast to collagenases from other species (human and sheep) that we work with in this laboratory, the murine enzyme was found to be destroyed by the freezing of supernatants, and this necessitated storage of material at 4°C, at which temperature it remained stable for over 6 months.

### *Comparison of collagenase output by tumours of high and low pulmonary-colonization potential and by non-neoplastic mammary tissue*

The collagenase activity secreted by each tumour or control group at each harvest period was used to plot the graphs showing the time course of collagenase release.

In addition, a general value for the collagenase secretory potential of each tumour was computed by dividing the accumulated output for each tumour by the number of days of harvesting and by the wet weight of tissues to produce a value of activity/day/0·5 g, as provided in Table II. These values were used for statistical comparison of collagenase output by the three experimental groups.

### *Active collagenase*

The mean output of active collagenase by HCP, LCP and controls is shown in Fig. 3a. It is evident that the tumours capable of heavy pulmonary colonization secrete much more of this enzyme than weak colonizers or non-neoplastic tissue (which cannot colonize the lung at all;

FIG. 3.—Comparison of means of (a) active collagenase, (b) latent collagenase and (c) total collagenase output (collagenase units/0·5 g/day) between the tumour and control groups. HCP = high colonization potential tumours, LCP = low colonization potential tumours, and CON = control tissues. Bars represent 95% confidence limits (data in Table II).

Price *et al.*, 1982). These results are statistically highly significant. The Kruskall–Wallis test confirms that the three groups are not drawn from a uniform population ($P < 0{\cdot}01$) and pairwise comparisons between selected groups are, therefore, legitimate. HCP differ from LCP with $P < 0{\cdot}005$, and from controls with $P < 0{\cdot}001$ (Wilcoxon rank-sum test). LCP do not differ significantly from controls, and if these groups are pooled, the tumours capable of heavily colonizing the lung differ very significantly ($P < 0{\cdot}005$) from weak or non-colonizers in production of active collagenase.

*Total collagenase*

The mean values for total collagenase activity in the supernatants are shown in Fig. 3b. As with the active form of the enzyme, the HCP tumours appear to secrete more than the other groups. Statistical analysis of the results for total collagenase production confirms that the three groups are different (Kruskall–Wallis Test, $P < 0{\cdot}001$) but pairwise comparisons do not show very significant differences between HCP and LCP colonizers ($0{\cdot}01 > P > 0{\cdot}05$) though both tumour groups are very significantly different from controls (Wilcoxon rank-sum test, HCP and LCP, both $P < 0{\cdot}001$).

### *Latent collagenase*

Latent-collagenase values are derived by subtraction of active from total enzyme, and not by direct measurement. They are therefore not independent variables and the results can be anticipated from the foregoing calculations. However, the means for latent-collagenase production by the three groups are shown in Fig. 3c and statistical tests confirm that the groups are different ($P < 0{\cdot}001$), and that the two tumour groups differ significantly from controls (both $P < 0{\cdot}001$) but not from each other.

### *Time-course of collagenase release in culture*

The mean collagenase output of the high, low and control groups at each harvest, is plotted against time in Fig. 4 for active collagenase and Fig. 5 for total collagenase. It is seen that enzyme output is much greater throughout the culture period in HCP than in LCP or non-neoplastic glands.

FIG. 4.—Time course for output of *active* collagenase over the entire culture period. The average collagenase output for each experimental group (HCP, □; LCP, ●; and CON, △) has been plotted against the average time for each harvest. Bars represent 95% confidence limits.

FIG. 5.—As Fig. 4, for *total* collagenase.

For active collagenase there is an initial dip in secretion which is particularly pronounced in the HCP group, after which there is a gradual rise, peaking at about the 10th day in culture.

### *Plasminogen-activator secretion*

It was also found that murine mammary neoplasms secrete PA and the mean output/day/0·5 g tissue is shown, along with their colonization potential in Table II. Statistical analysis of this information confirms that the three groups are significantly different ($P = 0{\cdot}005$) but here the HCP and LCP neoplasms are not very different from one another, though both secrete more enzyme than the controls ($P < 0{\cdot}001$). The mean values of PA production of all three groups are shown in Fig. 6. The breakdown of fibrin by the agent in the supernatant was confirmed to be plasminogen-dependent in all assays.

The time course of PA production by tumours (Fig. 7) is similar to that of collagenase. There is a dip at 5 days, with a subsequent peak at 10 days, after which enzyme production declines.

FIG. 6.—Comparison of plasminogen activator between HCP, LCP and CON. Bars represent 95% confidence limits (data from Table II).

FIG. 7.—Time course for output of plasminogen activator over the entire harvest period. Bars represent 95% confidence limits. See legend to Fig. 4 for details of graphic presentation.

### DISCUSSION

The above findings constitute unequivocal evidence that spontaneous mammary tumours in the mouse secrete a typical mammalian collagenase, and that tumours with high pulmonary colonization capability (HCP) produce significantly more of this enzyme *in vitro* than those of low potential or non-neoplastic controls. This is particularly obvious for active collagenase (Fig. 3a) and suggests involvement of this enzyme in tumour dissemination. However, it is important to comment that there is no direct evidence from this work that collagenase secretion *in vivo* is actually higher in HCP than in LCP tumours. The level of output *in vitro* indicates the secretory *capability* but does not necessarily reflect the *actual* secretion in the body. The association between HCP and collagenase output by these tumours *in vitro* is therefore not on its own conclusive proof of a causal role of the enzyme in metastatic spread. It is also appropriate to mention that high collagenase output is not a property unique to tumours. The enzyme is secreted in large quantities in other pathological conditions such as rheumatoid arthritis (Harris, 1978; Woolley *et al.*, 1980) and in certain non-pathological processes such as post-partum uterine involution (Woessner, 1980) and re-modelling of bone (Vaes, 1980). Nevertheless, the evidence in this investigation of a strong association between capacity for active collagenase production and metastatic colonization potential indicates that the enzyme is implicated in colonization after blood-borne dissemination and, more generally, that disordered regulation of the synthesis of this enzyme is an important factor predisposing to tumour spread.

The levels of total (*i.e.* active plus latent) collagenase production were not so significantly related to tumour colonization potential, but it is noteworthy that the accurate measurement of this variable is a capricious and difficult matter, depending on mode of activation (*e.g.* by trypsin or by organo-mercurials such as APMA) and on other as yet more intangible factors such as auto-activation (Stricklin *et al.*, 1977, 1978). To avoid diversionary forays into this controversial field, we chose a standard method of activation common to many publications on measurement of the latent enzyme. Had we used an activation protocol standardized by titration experiments with each batch of trypsin on our own material, the differences between the low and high colonizers might have been even more significant.

The specificities of the enzymes found for various collagen types are yet to be explored. It should be noted that this work has been conducted entirely on Type I collagen obtained from rat tail tendon. Although we have found by gel electrophoresis that the tumour supernatants also contain an agent capable of lysing Type IV collagen (Shields *et al.* in preparation), we do not yet have quantitative information on the secretion of other collagenases by these tumours (as reported by Liotta *et al.* (1979) for a transplantable neoplasm).

The output of plasminogen activator, though greater in neoplasms than in controls, did not bear any significant relationship to colonizing ability. We also found no association between amounts of PA and latent collagenase in the supernatants, and find no support in our system for the idea that PA may be one of the physiological activators of collagenase. Only free PA was measured, and it is possible that the levels of the cell-bound form of this enzyme might have been related to collagenase activity. However, attempts to activate samples of known high latent-collagenase content directly by deliberate addition of excess plasminogen and urokinase were not successful (data not shown). The physiological role of PA produced in raised quantities in many tumours and tumour-cell cultures is, therefore, not illuminated by these experiments.

It is not yet known which cell type produces the collagenase found in the HCP tumours, but morphological observations (Tarin, 1969) suggest that epithelial cells are involved, directly or by stimulation of other cell types within the tumour's It is hoped that immunocytochemical studies will eventually locate the site of production and secretion of this enzyme.

The immediate pathological implication of these findings is that they link the presence of the enzyme collagenase (already known to be detectable in many tumours) to the destructive and colonizing capabilities of spontaneous tumours *in vivo*. They also indicate a plausible mechanism for the morphological observation of removal of intercellular materials in primary and secondary tumour infiltration and expansion (Tarin, 1972, 1976) and this interpretation is supported by the immunocytochemical localization of the enzyme at the tumour–stromal interface (Woolley & Grafton, 1980).

We are very grateful to Dr S. A. Cederholm-Williams, Department of Haematology, John Radcliffe Hospital, Oxford, for gifts of purified human plasminogen, Dr D. E. Wooley, University Hospital of South Manchester, for a gift of partially purified human collagenase used as a positive standard, and to Dr J. F. Bithell, Department of Biomathematics (University of Oxford) for mathematical advice. We also wish to thank Miss L. D. Jones for technical assistance, Mrs P. Messer for help in preparation of the manuscript and Dr G. Wirl of the Institute of Molecular Biology, Salzburg, Austria, for valuable discussions and comments.

The work was financed by the Cancer Research Campaign of Great Britain, whose support is appreciated.

### REFERENCES

ABRAMSON, M., SCHILLING, R. W., HUANG, C. & SALOME, R. G. (1975) Collagenase activity in epidermoid carcinoma of the oral cavity and larynx. *Ann. Otol.*, **84**, 158.

DABBOUS, M. K., ROBERTS, A. N. & BRINKLEY, B. (1977) Collagenase and neutral protease activities in cultures of rabbit VX-2 carcinoma. *Cancer Res.*, **37**, 3537.

DRESDEN, M. H., HEILMAN, A. & SCHMIDT, J. D.

(1972) Collagenolytic enzymes in human neoplasms. *Cancer Res.*, **32**, 993.

EISEN, A. Z., JEFFREY, J. J. & GROSS, J. (1968) Human skin collagenase. Isolation and mechanism of attack on the collagen molecule. *Biochim. Biophys. Acta*, **151**, 637.

GISSLOW, M. T. & MCBRIDE, B. C. (1975) A rapid sensitive collagenase assay. *Anal. Biochem.*, **68**, 70.

GROSS, J. (1958) Studies on the formation of collagen. I. Properties and fractionation of neutral salt extracts of normal guinea-pig connective tissue. *J. Exp. Med.*, **107**, 247.

HARRIS, E. D., JR. (1978) Role of collagenases in joint destruction. In *Joints and Synovial Fluid*, Vol. 1 (Ed. Sokoloff.) New York: Academic Press. p. 243.

HASHIMOTO, K., YAMANISHI, Y., MAEYENS, E., DABBOUS, M. K. & KANZAKI, T. (1973) Collagenolytic activities of squamous-cell carcinoma of skin. *Cancer Res.*, **33**, 2790.

JONES, P. A., LAUG, W. E. & BENEDICT, W. F. (1975) Fibrinolytic activity in a human fibrosarcoma cell line and evidence for the induction of plasminogen activator secretion during tumor formation. *Cell*, **6**, 245.

KUETTNER, K. E., SOBLE, L., CROXEN, R. L. & MARCZYNSKA, B. (1977) Tumor cell collagenase and its inhibition by a cartilage-derived protease inhibitor. *Science*, **196**, 653.

LIOTTA, L. A., ABE, S., ROBEY, P. G. & MARTIN, G. R. (1979) Preferential digestion of basement membrane collagen by an enzyme derived from a metastatic murine tumor. *Proc. Natl Acad. Sci.*, **76**, 2268.

LIOTTA, L. A., TRYGGVASON, K., GARBISA, S., HART, I., FOLTZ, C. M. & SHAFIE, S. (1980) Metastatic potential correlates with enzymatic degradation of basement membrane collagen. *Nature*, **284**, 67.

MCCROSKERY, P. A., RICHARDS, J. F. & HARRIS E. D. (1975) Purification and characterization of a collagenase extracted from rabbit tumours. *Biochem. J.*, **152**, 131.

MARKUS, G., TAKITA, H., CAMIOLO, S. M., CORASANTI, J. G., EVERS, J. L. & HOBIKA, G. H. (1980) Content and characterization of plasminogen activators in human lung tumors and normal lung tissue. *Cancer Res.*, **40**, 841.

NAGY, B., BAN, J. & BRDAR, B. (1977) Fibrinolysis associated with human neoplasia: Production of plasminogen activator by human tumours. *Int. J. Cancer*, **19**, 614.

NICHOLSON, G. L., WINKELHAKE, J. L. & NUSSEY, A. C. (1976) An approach to studying the cellular properties associated with metastasis: Some *in vitro* properties of tumor variants selected *in vivo* for enhanced metastasis. In *Fundamental Aspects of Metastasis* (Ed. Weiss). Amsterdam: North-Holland. p. 291.

PRICE, J. E., CARR, D., JONES, L. D., MESSER, P. & TARIN, D. (1982) Experimental analysis of factors affecting metastatic spread using naturally-occurring tumours. In *Invasion and Metastasies.* (in press).

RIFKIN, D. B., LOEB, J. N., MOORE, G. & REICH, E. (1974) Properties of plasminogen activators formed by neoplastic human cell cultures. *J. Exp. Med.*, **139**, 1317.

SAKAI, T. & GROSS, J. (1967) Some properties of the products of reaction of tadpole collagenase with collagen. *Biochemistry*, **6**, 518.

SHIELDS, S. E., OGILVIE, D. J. & TARIN, D. (1982) In preparation.

STRICKLIN, G. P., BAUER, E. A., JEFFREY, J. J. & EISEN, A. Z. (1977) Human skin collagenase: Isolation of precursor and active forms from both fibroblast and organ cultures. *Biochemistry*, **16**, 1607.

STRICKLIN, G. P., EISEN, A. Z., BAUER, E. A. & JEFFREY, J. J. (1978) Human skin fibroblast collagenase: Chemical properties of precursor and active forms. *Biochemistry*, **17**, 2331.

TARIN, D. (1967) Sequential electron microscopical study of experimental mouse skin carcinogenesis. *Int. J. Cancer*, **2**, 195.

TARIN, D. (1969) Fine structure of murine mammary tumours: The relationship between epithelium and connective tissue in neoplasms induced by various agents. *Br. J. Cancer*, **23**, 417.

TARIN, D. (1972) Morphological studies on the mechanism of carcinogenesis. In *Tissue Interactions in Carcinogenesis* (Ed. Tarin). London: Academic Press. p. 227.

TARIN, D. (1976) Cellular interactions in neoplasia. In *Fundamental Aspects of Metastasis* (Ed. Weiss). Amsterdam: North-Holland. p. 151.

TARIN, D. & PRICE, J. E. (1979) Metastatic colonization potential of primary tumour cells in mice. *Br. J. Cancer*, **39**, 740.

TUCKER, W. S., KIRSCH, W. M., MARTINEZ-HERNANDEZ, A. & FINK, L. M. (1978) *In vitro* plasminogen activator activity in human brain tumors. *Cancer Res.*, **38**, 297.

VAES, G. (1980) Collagenase, lysosomes and osteoclastic bone resorption. In *Collagenase in Normal and Pathological Connective Tissues* (Eds. Woolley & Evanson). Chichester: John Wiley. p. 185.

WANG, B. S., MCLOUGHLIN, G. A., RICHIE, J. P. & MANNICK, J. A. (1980) Correlation of the production of plasminogen activator with tumor metastasis in B16 mouse melanoma cell lines. *Cancer Res.*, **40**, 288.

WIRL, G. & FRICK, J. (1979) Collagenase: A marker enzyme in human bladder cancer? *Urol. Res.*, **7**, 103.

WOESSNER, J. F., JR. (1980) Collagenase in uterine resorption. In *Collagenase in Normal and Pathological Connective Tissues* (Eds. Woolley & Evanson). Chichester: John Wiley. p. 223.

WOOLLEY, D. E., GLANVILLE, R. W., ROBERTS, D. R. & EVANSON, J. M. (1978) Purification, characterization and inhibition of human skin collagenase. *Biochem. J.*, **169**, 265.

WOOLLEY, D. E., TETLOW, L. C. & EVANSON, J. M. (1980) Collagenase immunolocalization studies of rheumatoid and malignant tissues. In *Collagenase in Normal and Pathological Connective Tissues* (Eds. Woolley & Evanson). Chichester: John Wiley. p. 105.

WOOLLEY, D. E. & GRAFTON, C. A. (1980) Collagenase immunolocalization studies of cutaneous secondary melanomas. *Br. J. Cancer*, **42**, 260.

YAMANISHI, Y., DABBOUS, M. K. & HASHIMOTO, K. (1972) Effect of collagenolytic activity in basal cell epithelioma of the skin on reconstituted collagen and physical properties and kinetics of the crude enzyme. *Cancer Res.*, **32**, 2551.

YAMANISHI, Y., MAEYENS, E., DABBOUS, M. K., OHYAMA, H. & HASHIMOTO, K. (1973) Collagenolytic activity in malignant melanoma: Physicochemical studies. *Cancer Res.*, **33**, 2507.